# Fungal Endophytes of *Vitis vinifera—*Plant Growth Promoters or Potentially Toxinogenic Agents?

**DOI:** 10.3390/toxins14020066

**Published:** 2022-01-19

**Authors:** Milena Stranska, Zbynek Dzuman, Nela Prusova, Adam Behner, Irena Kolouchova, Petra Lovecka, Tomas Rezanka, Miroslav Kolarik, Jana Hajslova

**Affiliations:** 1 Department of Food Analysis and Nutrition, University of Chemistry and Technology, Prague, Technicka 3, 166 28 Prague, Czech Republic; zbynek.dzuman@vscht.cz (Z.D.); Nela.Prusova@vscht.cz (N.P.); adam.behner@vscht.cz (A.B.); jana.hajslova@vscht.cz (J.H.); 2 Department of Biotechnology, University of Chemistry and Technology, Prague, Technicka 3, 166 28 Prague, Czech Republic; irena.kolouchova@vscht.cz; 3 Department of Biochemistry and Microbiology, University of Chemistry and Technology, Prague, Technicka 3, 166 28 Prague, Czech Republic; petra.lovecka@vscht.cz; 4 Institute of Microbiology of the Czech Academy of Sciences, Videnska 1083, 142 20 Prague, Czech Republic; rezanka@biomed.cas.cz (T.R.); mkolarik@biomed.cas.cz (M.K.)

**Keywords:** microscopic filamentous fungi, endophytes, mycotoxins, liquid chromatography, mass spectrometry

## Abstract

Fungal endophytes occurring in grapevine (*Vitis vinifera* L.) are usually important sources of various compounds with biological activities with great potential for use in agriculture. Nevertheless, many species isolated from this plant belong to the genera *Fusarium*, *Alternaria*, or *Aspergillus,* all of which are well-known to produce mycotoxins. Our study is focused on the assessment of the toxinogenic potential of fungal endophytes isolated from vineyards in the Czech Republic. In total, 20 endophytic fungal species were cultivated in wine must, and 57 mycotoxins of different classes were analysed by liquid chromatography coupled with mass spectrometry. As a result, alternariol, tentoxin, meleagrin, roquefortine C, gliotoxin, and verruculogen were detected in the culture medium, of which verruculogen followed by gliotoxin were the most frequent (present in 90 and 40% of samples, respectively) and most concentrated (up to thousands ng/mL). The alternaria mycotoxins alternariol and tentoxin were detected not only in *Alternaria* sp. cultures, but traces of these mycotoxins were also quantified in the *Diatripe* and *Epicoccum* cultures. Meleagrin and roquefortine C were detected in *Didymella sancta* and *Penicillium crustosum,* gliotoxin was detected in *Alternaria* sp., *Didymella* sp., *Aureobasidium pullulans*, *Cladosporium herbarum*, *Penicillium crustosum* and *Pleurophoma ossicola*, and verruculogen was quantified in 99% of endophytic isolates investigated. The potential of endophytes to produce mycotoxins should be carefully checked, specifically in cases where they are intended for the purpose of *V. vinifera* growing.

## 1. Introduction

Endophytes are classified as a family of microorganisms residing intracellularly in plant tissues. Cultivated plants, as well as plant species growing in unexplored areas of the world, host different endophytes that play an important role in biodiversity of the whole ecosystem. Despite most endophytes being represented by bacteria, a significant proportion of internal plant microbiota belong to classes of fungal endophytes.

Currently, endophytic microscopic fungi are well known as important sources of bioactive compounds produced as secondary metabolites, including benzopyranones, flavonoids, phenolic acids, quinones, steroids, terpenoids, and/or alkaloids, that possess biological activities of medicinal importance. In addition, plant hormones such as gibberellins, jasmonates, abscisic acid, and many others, play a notable role in plant growth, promoting and protecting against biotic and abiotic stress [1,2]. Specifically, endophytes producing the latter group of compounds represent a promising source of microorganisms with potential to be used in agriculture as plant growth supporters and biological control agents, which is in line with current needs and trends for the sustainable production of eco-friendly crops [3]. Talking about the agro-biotechnological potential of fungal endophytes in the breeding of agricultural crops, their significant phytoremediation potential should also be mentioned [1]. Strategies for the application of endophytes in agricultural systems as inoculants of the soil, seed dressings or inocula for continuous application to crops during planting have been developed [3].

Over the last decades, intensive research has been undertaken focusing on bioprospecting of endophytic microbiota of the grapevine (*Vitis vinifera* L.), as one of the most economically important crops. Special attention has been paid to the positive effects of endophytes in fighting various grapevine diseases, e.g., grapevine downy mildew caused by *Plasmopara viticola*, gray rot of grapes associated with *Botrytis cinerea* [4,5,6,7], or various grapevine trunk diseases [8]. Other papers have reported on the effects of endophytes on the production of health-promoting compounds [9], or of the tuning of the sensory profile [10]. As mentioned above, considering the current trends towards reducing the usage of chemicals in agriculture, the investigation of this aspect is very fashionable. However, a number of biocontrol agents, presented by authors as “promising” in terms of reducing various fungal diseases, belong to toxinogenic species, e.g., *Alternaria alternata* or *Fusarium proliferatum* [4,5,6,7] generally known to produce a wide spectrum of toxic secondary metabolites—mycotoxins. The potential of endophytic fungi to act as fine-tune regulators in the synthesis of bioactive secondary metabolites and the modulation of sensory quality in grapes is another example of the role of endophytes in plant production. In the study of Yang et al., a *Fusarium* fungal strain was presented as one of the most promising endophytic candidates [10], but the authors did not discuss its well-known ability to produce various classes of fusarium mycotoxins, which may significantly limit the potential of this endophytic species. In another study, the ability of endophytes to produce the antioxidant resveratrol was addressed, and, *Aspergillus*, *Penicillium* and *Alternaria* species were identified as the most productive, highlighting *Alternaria* strains as the most potent and stable [9]. However, again, the potential of *Aspergillus*, *Penicillium* and *Alternaria* fungi to produce mycotoxins was not considered. Notwithstanding the biotechnological potential of endophytes and their possible application in grapevine cultivation, especially for industrial scale production, the toxinogenic potential of particular fungal endomicrobiota should not be neglected.

With regard to the above indicated lack of knowledge on mycotoxins produced by endophytes, the aim of our study was to investigate the potential of endophytic fungi isolated from vineyards in the Czech Republic to produce these toxic secondary metabolites. Fungal endophytes were isolated from various parts of *V. vinifera* plants and cultivated in the wine must medium. Altogether, 57 mycotoxins commonly produced by *Fusarium*, *Alternaria*, *Aspergillus*, *Penicillium*, and other fungal species were analysed by ultra-high performance liquid chromatography coupled with high resolution tandem mass spectrometry (U-HPLC-HRMS/MS). To our knowledge, this is the first paper describing the potential of *V. vinifera*-residing fungal endophytes to produce mycotoxins using a sensitive and fully validated method.

## 2. Results

### 2.1. Validation of U-HPLC-HRMS/MS for Mycotoxins Analysis

Full validation of U-HPLC-HRMS/MS for analysis of all 57 mycotoxins in the wine must was performed, and validation characteristics are presented in Table 1. The lowest calibration levels (LCL) for particular mycotoxins were 0.5 ng/mL (for 72% of analytes), and 92% of all mycotoxins analysed had a quantification limit from 0.5 to 5 ng/mL. Higher LCLs were observed for the polar early eluting analytes nivalenol, patulin, deoxynivalenol-3-glucoside, and for tenuazonic and penicillic acid. As for matrix effects, for the vast majority of analytes, matrix-induced ionization suppression was observed; however, the degree of signal suppression was not significant. The reason of the moderate matrix effects was removal of sugars and other polar substances from the aqueous-acetonitrile extract after addition of inorganic salts, centrifugation and partitioning of originally miscible solvents, where the polar compounds preferred to remain in the bottom aqueous layer. Recoveries of all analytes were in the range of 68–118%, with the exception of deoxynivalenol-3-glucoside (34.5% due to its high polarity, thus less effective transfer into the acetonitrile layer during extraction). The relative standard deviations (RSD) ranged from 1.1% to 6.9% for the spiking level of 50 ng/mL and 2.2% to 9.7% for the spiking level of 10 ng/mL, which is in line with guidance usually considered for assessment of quality parameters of multi-toxin methods [11].

### 2.2. Fungal Endophytes Isolated from Grapevine

From twenty different endophytic fungal species isolated from various parts of *V. vinifera* plants, three of them were of the genus *Aspergillus* (*Aspergillus fumigatus*, *Aspergillus niger* and *Aspergillus pseudodeflectus*), two of them belonged to *Alternaria* genus (*Alternaria arborescens* and *Alternaria astroemeriae*), another two belonged to the genus *Cladosporium* (*Cladosporium cladosporioides* and *Cladosporium herbarum*), and two were of *Didymella* genus (*Didymella negriana* and *Didymella sancta*). The other fungal endophytes were identified as *Aureobasidium pullulans*, *Dendrophoma juglandina*, *Diatrype stigma*, *Epicoccum nigrum*, *Lophiostoma corticola*, *Neosetophoma shoemakeri*, *Penicillium crustosum*, *Phaeosphaeriaceae* sp., *Pleurophoma ossicola*, *Pseudogymnoascus pannorum*, and *Sporocadus rosigena*. Details of their microbiological characteristics and plant parts from which these endophytic fungi were isolated are shown in Table 2.

**Figure 1:**
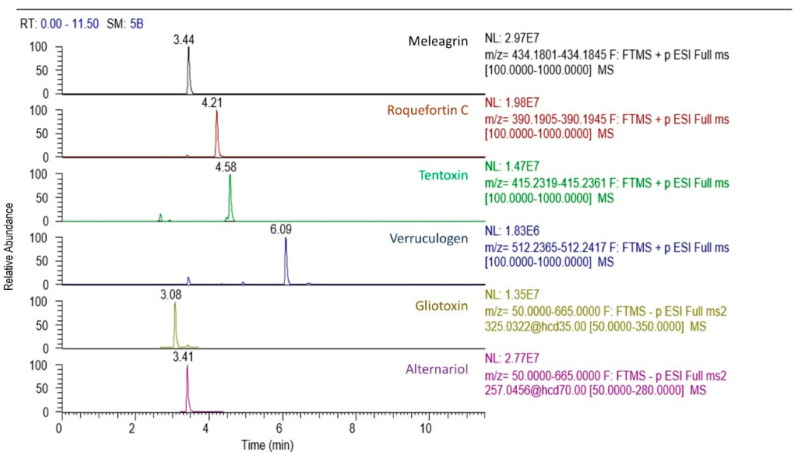
The U-HPLC-HRMS/MS chromatogram of selected mycotoxins in matrix-matched standard (50 ng/mL).

**Figure 2:**
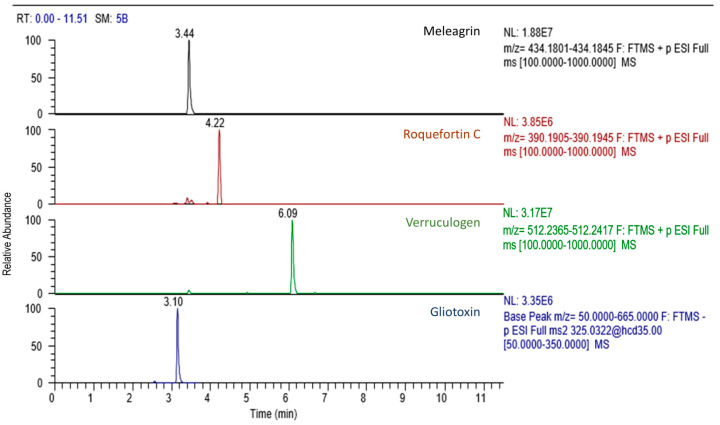
The U-HPLC-HRMS/MS chromatogram of mycotoxins in the *Didymella sancta* culture.

In the wine must cultures, the mycotoxins alternariol, tentoxin, and prenylated indole alkaloids meleagrin, roquefortine C, gliotoxin, and verruculogen were detected (see Table 3). Although alternariol is predominantly produced by micromycetes of the genus *Alternaria*, traces of this mycotoxin were detected in *Diatrype stigma* and *Epicoccum nigrum*. Low concentrations of the mycotoxin tentoxin, (also commonly produced by *Alternaria* genus), were detected in the *Alternaria astroemeriae* endophyte. Mycotoxins meleagrin and roquefortine C were detected in strains of *Didymella sancta* and *Penicillium crustosum*. A considerably higher frequency of occurrence and several orders of magnitude higher concentrations of gliotoxin were determined. The isolates *Pleurophoma ossicola*, *Cladosporium herbarum*, *Alternaria arborescens* and *Penicillium crustosum* produced hundreds to thousands of ng/mL, but lower amounts of gliotoxin were detected also in *Aureobasidium pullulans*, *Didymella sancta*, *Didymella negriana* and *Alternaria astroemeriae* cultures. The highest frequency of occurrence and highest concentrations were observed for the mycotoxin verruculogen, the presence of which was demonstrated in almost all endophytic isolates in the order of tens up to thousands of ng/mL. Figure 1 shows chromatographic peaks of standards of mycotoxins that were positive in the samples, and Figure 2 depicts example of chromatogram of mycotoxins present in *Didymella sancta* culture.

## 3. Discussion

Generally, the presence of mycotoxins in agricultural crops and exposure to consumers is a global problem carrying substantial and widespread threats to health [12]. Contrary to other plants, where the toxinogenic potential of fungal endophytes have been studied (predominantly leaf vegetable [13], legumes [14] or different grasses and plants potentially toxic for grazing livestock [15,16,17]), in the case of *V. vinifera*, this important aspect of grapevine endomicrobiota remained unstudied.

Most of the mycotoxins detected in our study belong to the prenylated indole alkaloids, bioactive toxins mostly produced by *Penicillium* and *Aspergillus* fungal species [18,19]. The mycotoxin produced by a majority of endophytic *V. vinifera* fungal species isolated and cultivated in our study was verruculogen, a newly emerging mycotoxin showing cytotoxicity and apoptotic effects on human cells [20]. Our results confirm that quantities of verruculogen produced were the highest in *Penicillium* and *Aspergillus* isolates (concentrations were higher than 1000 µg/L). Nevertheless, several other fungal species also produced this mycotoxin in rather high concentrations close to that previously described (i.e., *Alternaria arborescens*, *Aureobasidium pullulans*, or *Didymella sancta*, see Table 3). The second most frequent and concentrated mycotoxin from the indole alkaloids group was gliotoxin, previously reported to be produced by *Aspergillus fumigatus*, *Eurotium chevalieri*, *Trichoderma virens*, *Neosartorya pseudofischeri* and some *Penicillium* and *Acremonium* species [21]. According to our results, the most potent producers were *Pleurophoma ossicola*, *Cladosporium herbarum* and *Alternaria arborescens*, with hundreds up to thousands µg/L in cultures from the wine must. Both roquefortine C and meleagrin, usually produced by many *Penicillium* species [22], were detected in strains of *Penicillium crustosum*, but also *Didymella sancta*. Despite roquefortine C being previously reported to be produced by *Penicillium crustosum* [22], production of meleagrin by this fungal species was shown for the first time. It should be noted that most of the verruculogen, gliotoxin, meleagrin and roquefortine C-producing species identified in our study have not been reported previously to produce these mycotoxins. In addition to the four mycotoxins discussed above, other prenylated indole alkaloids such as ergot alkaloids or cyclopiazonic acid were included in our U-HPLC-HRMS/MS method and, despite low detection limits, their presence was not demonstrated in the cultures. The mycotoxins alternariol and tentoxin, usually produced by *Alternaria alternata* as the most common alternaria mycotoxin-producing species [23], were quantified at relatively trace levels in *Alternaria astroemeriae*, *Diatrype stigma* and *Epicoccum nigrum* isolates. Again, these fungal strains were not previously reported to produce altertoxins. Alternariol monomethyl ether and tenuazonic acid, as other altertoxins included in the analytical method, were not shown to be produced by any of the endophytic fungal isolates investigated.

Among current strategies for sustainable intensive agriculture, the use of endophyte-based preparations definitely has its place. Nevertheless, as long as endophytes are used as plant growth promotors in routine cultivation of grapevines (similarly as in the case of commercially available preparations AR1, AR37 and Endo5, containing fungal endophytes protecting ryegrass against the Argentine stem weevil [3,24,25]), their potential to produce toxic secondary metabolites must be carefully checked. Despite the fact that under field conditions of the vineyard, mutually beneficial relationships between endophytes and plants may regulate mycotoxin production, this situation cannot, a priori, be assumed, and “worst-case-scenarios” should be considered. Firstly, endophyte-plant crosstalk cannot be fully predicted, and secondly, there is always a risk that fungal strains applied will establish as epiphytes, where mutually beneficial mechanisms between plant and microbiota do not work and mycotoxin production can take place readily. Moreover, as a result of recently much-discussed climate change, it is commonly found that in addition to grapevine traditional ochratoxin A, new (“emerging”) mycotoxins are starting to naturally appear in grapes and other agricultural crops [26,27,28] and, therefore, any intervention affecting the composition of fungal species in grapevine should be investigated properly.

## 4. Materials and Methods

### 4.1. Sampling of Grapevine and Endophytes Isolation/Cultivation

Endophytes were isolated from *V. vinifera* plants of Muller Thurgau (MT), Pinot Gris (PG), Pinot Noir (PN), and Riesling Rheinhessen (RR) that were collected from vineyards within the Czech Republic. Sampling of canes as lignified stems of plants (approximately 100 g) was performed in January, May, August and October 2019. Leaves were sampled (30 to 100 g depending on the sampling season) in May, August and October 2019. Berries were sampled (500 g) in September 2019. Samples were stored at −80 °C before further processing in the laboratory.

Before endophyte isolation, the plant material was surface sterilized by sequential immersion in 0.625% aqueous sodium hypochlorite with a droplet of Tween 80 (7 min), followed by 70% aqueous ethanol (3 min). After these procedures, samples were rinsed four times with sterile water (15 min). Surface-sterilized tissues were homogenized and used to inoculate YGC medium (yeast extract glucose chloramphenicol agar) and incubated at 20 °C for at least 72 h. Fungi displaying different morphologies were re-streaked on new plates to obtain clean cultures.

Fungal cultures on YPD (yeast extract peptone dextrose) agar slants were used as inoculum for the pre-cultures. The pre-cultures of fungal endophytes were further cultivated in the wine must (the sugar content of 20 g/L) in Erlenmeyer flasks (volume of media 50 mL) at 30 °C for 7 days. The spore concentration was adjusted by counting in Bürker chamber to 106 per mL. Until the U-HPLC-HRMS/MS analysis, the samples were stored in −18 °C.

### 4.2. Molecular Genetic Identification

Genomic DNA was isolated from pure microscopic fungal cultures using the ArchivePure DNA Yeast/Gram Positive Bacteria Kit (5 PRIME, Hamburg). Subsequently, the nuclear ribosomal ITS1-5,8S-ITS2 region was determined for all strains according to [29]. Due to the low resolution of the ITS region in some fungal genera, sequencing of other sections were carried out to clarify identification. Elongation factor 1 alpha (EF1α) was amplified and sequenced using primers EF-728F/EF-986R and EF1-983F/EF1-2218R according to [29]. The partial β-tubulin (TUB2) gene was amplified using T1/T2 according to [30]. PCR product were purified with ExoSAP Cleanup Reagent (Thermo Fisher Scientific, Waltham, MA, USA) and both strand sequenced at Macrogen Europe (Amsterdam, The Netherlands). The sequences obtained were manually cut from unreadable sections and the highest sequence probability was searched for in the GenBank database using BlastN similarity search. In particular sequences were compared with those from reference (e.g., type) strains published by taxonomic studies. Characterization of samples containing fungal endophytes is shown in Table 2.

### 4.3. Determination of Mycotoxins

#### 4.3.1. Chemicals 

Anhydrous magnesium sulphate (≥99.5%), sodium chloride (≥99.0%), methanol and acetonitrile (both LC–MS grade) were obtained from Merck (Prague, Czech Republic). Deionized water (18.2 MΩ) was obtained from a Milli-Q system (Merck-Millipore, MA, USA). Analytical standards of 57 mycotoxins, specifically 22 *Fusarium* toxins 3- and 15-acetyldeoxynivalenol, deoxynivalenol, deoxynivalenol-3-glucoside, diacetoxyscirpenol, fusarenon X, HT-2 toxin, neosolaniol, nivalenol, T-2 toxin, verrucarol, zearalenone, α- and β-zearalenol, fumonisins B1, B2 and B3, beauvericin, enniatins A, A1, B and B1; 17 *Aspergillus* and *Penicillium* toxins aflatoxins B1, B2, G1 and G2, ochratoxin A, patulin, sterigmatocystin, citrinin, cyclopiazonic acid, mycophenolic acid, penicillic acid, gliotoxin, meleagrin, paxilline, penitrem A, roquefortine C, verruculogen; 12 *Claviceps* alkaloids agroclavine, ergometrine, ergosine, ergosinine, ergotamine, ergotaminine, ergocornine, ergocorninine, ergocryptine, ergocryptinine, ergocristine, ergocristinine; four *Alternaria* toxins alternariol, alternariol monomethyl ether, tentoxin and tenuazonic acid; two *Stachybotrys* and *Phomopsis* toxins stachybotrylactam and phomopsin A, respectively, were purchased from Merck (Prague, Czech Republic), Romer Labs (Tulln, Austria), Cayman Chemical (Ann Arbor, MI, USA), Toronto Research Chemicals (North York, ON, Canada) and LKT Laboratories (St. Paul, MN, USA), for specific details, see Table S1 of the Supplementary Materials. The purity of analytical standards was in the range of 95.1–100%. The mixed standard in acetonitrile was prepared for further usage.

#### 4.3.2. Sample Preparation 

A 10 mL aliquot of wine must culture was pipetted into a centrifuge tube (50 mL) and handshaken with acetonitrile (10 mL, 2 min). Magnesium sulphate (4 g) and sodium chloride (1 g) were then added, followed by vigorous handshaking (1 min) and centrifugation (5 min, 13 081 g; Rotina 380R, Hettich, Germany). An extract from the upper acetonitrile layer was transferred into an amber glass vial and immediately analysed by U-HPLC-HRMS/MS.

#### 4.3.3. Instrumental Analysis 

Separation of mycotoxins was conducted on an ultra-high performance liquid chromatograph UltiMateTM 3000 (Thermo Scientific, Waltham, MA, USA) with analytical column Acquity UPLC^®^ HSS T3 (100 × 2.1 mm, 1.8 µm; Waters, Milford, MA, USA) held at 40 °C. Autosampler temperature was 10 °C and injection volume was 3 µL. Different mobile phases and elution gradients were used for analytes providing higher ionization yield in positive and negative modes of electrospray (ESI+/ESI−). In ESI+, the run time was 12 min and mobile phases consisted of 5 mM ammonium formate and 0.2% formic acid both in water (A) and methanol (B). The gradient started at 10% of B and 0.3 mL/min, changing to 50% of B in 1 min, and another change to 100% of B in next 8 min with flow rate simultaneously increased to 0.4 mL/min. The analytical column was then washed for 2 min with 100% B at 0.4 mL/min and reconditioned for 2 min with the initial mobile phase. In ESI−, 5 mM ammonium acetate in water (C) and neat methanol (D) were used. The gradient started at 10% of D and 0.3 mL/min followed by a steep change to 50% of D at 1 min and 0.3 mL/min and then a gradual change to 100% of D at 6.5 min with the mobile phase flow rate increased to 0.4 mL/min. The analytical column was washed for 2 min at 100% of D at 0.4 mL/min and reconditioned for 2 min with the initial composition of mobile phases.

Detection of analytes was performed using high resolution tandem mass spectrometer Q-ExactiveTM Plus (Thermo Scientific, Waltham, MA, USA) equipped with HESI II interface and quadrupole-orbitrap mass analyzers. The ion source parameters were as follows: sheath/auxiliary gas flow rate 45/10 arbitrary units, heater temperature 300 °C, S-lens RF level 55, capillary voltage ± 3.5 kV and capillary temperature 320 °C. Detection of mycotoxins in ESI+ was in full spectral acquisition mode with data dependent MS2 (fullMS-ddMS2), enabling acquisition of the MS/MS spectra from the particular primary ion that is defined in the analytes ‘target list’ (at least one MS/MS spectra per peak are acquired for confirmatory purposes). In this detection mode, the m/z of the primary ion was used for quantification, and the MS/MS spectra confirmed the analytes. Parameter settings of the ESI+ method were as follows: (i) full MS: resolution 70,000 full width at half maximum (FWHM), mass range 50–1000 *m*/*z*, automatic gain control (AGC target) 3 × 10^6^ and maximum inject time (maxIT) 100 ms, and (ii) ddMS2: resolution 17,500 FWHM, mass range 50 up to *m*/*z* of particular analyte, AGC target 1 × 10^5^, maxIT 50 ms, loop count 10 and isolation window width 1 *m*/*z*. For ESI−, the data acquisition was performed in a parallel reaction monitoring (PRM) mode, enabling full time acquisition of product ions (MS/MS spectra) in the defined retention time window (60 s), and for quantification the most intensive product ion is used (this approach assures better selectivity and better detectability for some ESI+ ionizing early eluting analytes). The conditions were as follows: resolution 17,500 FWHM, mass range 50 up to *m*/*z* of particular analyte, AGC target 1 × 10^5^, maxIT 50 ms. Overview of retention times, exact masses of precursor and product ions of target analytes together with optimal normalized collision energies (NCE) are summarized in Table S2 of the Supplementary material. Xcalibur 4.2 (Thermo Scientific, Waltham, MA, USA) was used to calculate the exact masses, control the LC–MS system and data evaluation.

#### 4.3.4. U-HPLC-HRMS/MS Method Validation and Mycotoxins Quantification 

Mycotoxins were quantified using matrix-matched calibration standards. The calibration batch was prepared in the range 0.1–200 ng/mL, with calibration points 0.1; 0.2; 0.5; 1; 5; 10; 20; 50; 100; 200 ng/mL. Appropriate volumes of mixed standard (1000 or 100 ng/mL) was added into the vials, acetonitrile was evaporated under a gentle nitrogen stream, and 1 mL of wine must medium, prepared according to the above described sample preparation procedure, was added (the wine must was previously checked to be free of mycotoxins). If the signal of analyte in the sample exceeded the highest calibration point, an appropriate dilution was performed. All concentrations of positive analytes were corrected for recoveries.

Recoveries and repeatabilities of analytes were verified by spiking the calculated volume of mixed standard (1000 ng/mL) to 10 mL of culture No 13 (*Epicoccum nigrum*) as a sample with minimal mycotoxin contamination (the sample with the lowest mycotoxins contamination was selected after the U-HPLC-HRMS/MS pre-screening), to obtain final spiking concentrations of 50 and 10 ng/g. The spiking was performed in seven repetitions for each concentration level. Each ‘spike’ was further processed according to the procedure described in the sample preparation paragraph. Recoveries were calculated as relative rations of ‘determined’ to ‘spiked’ concentrations (arithmetic mean from 7 repetitions), and repeatabilities were calculated as relative standard deviations (RSDs, %) from these 7 repetitions.

The LCLs for particular analytes were determined as the lowest calibration points of matrix-matched calibration batch which was possible to repeatedly integrate during at least three independent measurements, each at least 14 days apart (as explained previously by [31], for high resolution mass spectrometry, the LCL definition is more suitable than the traditionally used limits of detection/quantification, LODs/LOQs).

To assess the degree of matrix induced ionization suppression/enhancement, a calibration batch in acetonitrile was prepared in the range of 0.1–200 ng/mL (calibration points 0.1; 0.2; 0.5; 1; 2; 5; 10; 20; 50; 100; 200 ng/mL, and the matrix effects (ME, %) were calculated as the matrix-matched calibration slope divided by the solvent calibration slope, multiplied by 100. The lowest calibration levels for particular mycotoxins were determined as the lowest concentrations of matrix-matched standards that it was possible to repeatedly determine over a longer time period. The full validation parameters of the U-HPLC-HRMS/MS method are presented in Table 1.

## Figures and Tables

**Table 1:** Validation parameters of the U-HPLC-HRMS/MS method employed for mycotoxins analysis in fungal cultures.

Mycotoxin	Rt [min] ^1^	LCL [ng/mL] (MeCN)	LCL [ng/mL] (Must)	Recovery [%] (50 ng/mL)	Recovery [%] (10 ng/mL)	RSD [%] (50 ng/mL)	RSD [%] (10 ng/mL)	ME [%] ^2^
15-Acetyldeoxynivalenol	2.69	0.1	0.5	87.0	87.3	6.2	8.6	75.1
3-Acetyldeoxynivalenol	2.57	0.2	0.5	84.8	97.4	4.5	5.9	86.1
Aflatoxin B1	3.45	0.1	0.5	78.9	83.7	5.6	6.9	68.8
Aflatoxin B2	3.26	0.1	0.5	81.4	84.1	2.9	6.2	72.2
Aflatoxin G1	3.01	0.1	0.5	84.2	86.6	3.4	7.1	68.7
Aflatoxin G2	2.85	0.1	0.5	84.6	84.7	3.0	7.3	60.7
Agroclavine	2.36	0.1	0.5	73.8	72.6	4.5	5.8	59.6
Alternariol	3.37	0.1	0.5	77.6	67.9	3.6	7.7	62.8
Alternariol monomethyl ether	4.03	0.1	0.5	79.1	71.7	2.2	6.6	79.9
Beauvericin	7.98	0.1	0.5	86.6	90.8	3.5	5.1	85.2
Citrinin	2.87	0.1	0.5	95.5	100	6.2	9.7	85.6
Cyclopiazonic acid	3.06	0.2	0.5	80.3	94.1	4.7	9.1	105
Deoxynivalenol	2.07	0.2	5	84.7	96.3	4.5	7.6	79.5
Deoxynivalenol-3-glucoside	1.98	5	10	34.5	-	5.1	-	62.3
Diacetoxyscirpenol	3.53	0.1	0.5	87.9	91.1	2.7	4.3	101
Enniatin A	8.36	0.1	0.5	81.3	83.0	1.7	2.2	93.9
Enniatin A1	8.20	0.1	0.5	85.4	91.5	3.2	6.1	87.3
Enniatin B	7.83	0.1	0.5	87.5	91.3	3.6	6.6	87.9
Enniatin B1	8.03	0.1	0.5	89.8	90.0	2.4	7.2	92.9
Ergocornine	3.27	0.1	0.5	79.6	86.3	6.1	8.7	82.8
Ergocorninine	3.81	0.1	0.5	82.5	85.9	3.4	7.4	86.0
Ergocristine	3.72	0.1	0.5	77.0	76.4	2.5	8.0	88.2
Ergocristinine	4.25	0.1	0.5	80.2	83.9	4.5	6.5	82.3
Ergocryptine	3.67	0.1	0.5	78.9	86.7	1.9	6.5	85.3
Ergocryptinine	4.14	0.1	0.5	82.1	85.8	3.0	7.1	76.4
Ergometrine	1.87	0.1	0.5	71.6	70.0	2.0	6.3	68.0
Ergosine	3.01	0.1	0.5	77.9	73.4	1.5	6.7	62.8
Ergosinine	3.10	0.1	0.5	80.4	81.4	2.9	6.6	77.6
Ergotamine	3.15	0.1	0.5	72.9	79.6	1.2	7.4	58.0
Ergotaminine	3.20	0.1	0.5	76.5	76.7	2.8	5.5	76.3
Fumonisin B1	4.37	5	5	104	118	3.6	7.3	133
Fumonisin B2	5.46	5	5	100	113	6.7	8.0	81.5
Fumonisin B3	4.99	5	5	99.4	106	2.0	7.9	80.2
Fusarenon X	2.23	5	5	80.9	-	1.3	-	94.7
Gliotoxin	3.04	0.5	1	81.1	68.6	1.9	6.3	82.7
HT-2 toxin	4.27	0.1	0.2	87.7	102	5.8	8.9	84.5
Meleagrin	3.35	0.1	0.5	90.0	89.2	4.5	5.1	80.5
Mycophenolic acid	4.68	0.1	0.5	87.0	92.0	2.9	8.4	108
Neosolaniol	2.30	0.1	0.5	82.7	96.0	3.3	7.7	79.6
Nivalenol	1.82	5	10	73.5	-	3.2	-	58.2
Ochratoxin A	5.34	0.1	5	87.3	88.6	1.1	4.3	99.1
Patulin	1.82	2	10	85.4	71.8	2.4	8.3	81.2
Paxilline	6.93	0.1	0.5	86.0	105	5.7	8.0	88.2
Penitrem A	2.08	0.5	5	92.8	113	3.9	7.6	73.8
Penicillic acid	4.42	10	20	74.4	-	3.2	-	52.1
Phomopsin A	2.60	1	2	87.1	84.0	3.2	6.0	104
Roquefortine C	4.10	0.1	0.5	83.2	91.9	4.1	6.7	70.0
Stachybotrylactam	6.31	0.1	0.5	88.8	88.9	4.5	6.0	92.5
Sterigmatocystin	5.68	0.1	0.5	76.5	80.3	3.5	6.2	85.4
T-2 toxin	4.89	0.1	0.5	85.6	90.8	3.6	7.3	83.0
Tentoxin	4.52	0.1	0.5	89.6	90.9	2.4	7.0	93.6
Tenuazonic acid	3.74	50	100	-	-	-	-	-
Verrucarol	2.53	0.2	0.5	84.2	86.0	5.1	6.5	61.0
Verruculogen	6.06	2.5	5	86.2	98.7	2.3	4.3	114
Zearalenone	3.85	0.1	0.5	91.3	91.8	2.3	5.4	82.3
α-Zearalenol	3.75	0.2	0.5	86.9	93.6	6.9	8.5	75.9
β-Zearalenol	3.53	0.2	0.5	89.3	93.6	5.6	6.8	88.0

^1^ Retention time, ^2^ ME of 100% means no matrix effect.

**Table 2:** Fungal endophytes isolated from particular *V. vinifera* plant parts.

Sampling Season	Grapevine Variety ^1^	Grapevine Plant Part	Endophyte Identified
winter	RR	canes	*Alternaria arborescens*
autumn	PN	leaves	*Alternaria astroemeriae*
summer	MT	leaves	*Aspergillus fumigatus*
winter	PN	canes	*Aspergillus niger*
winter	PN	canes	*Aspergillus pseudodeflectus*
spring	PN	canes	*Aureobasidium pullulans*
winter	MT	canes	*Cladosporium cladosporioides*
spring	PN	canes	*Cladosporium herbarum*
spring	PG	leaves	*Dendrophoma juglandina*
winter	MT	canes	*Diatrype stigma*
winter	MT	canes	*Didymella negriana*
autumn	PN	leaves	*Didymella sancta*
spring	MT	leaves	*Epicoccum nigrum*
summer	PG	leaves	*Lophiostoma corticola*
spring	RR	canes	*Neosetophoma shoemakeri*
autumn	MT	berries	*Penicillium crustosum*
spring	RR	canes	*Phaeosphaeriaceae sp.*
spring	RR	canes	*Pleurophoma ossicola*
spring	MT	canes	*Pseudogymnoascus pannorum*
spring	PG	leaves	*Sporocadus rosigena*

^1^ MT—Muller Thurgau, PG—Pinot Gris, PN—Pinot Noir, RR—Riesling Rheinhessen.

**Table 3:** Concentrations of mycotoxins in endophyte cultivates (ng/mL ± sd).

Sample No.	Endophyte Species Taxonomy	Alternariol	Tentoxin	Meleagrin	Roquefortine C	Gliotoxin	Verruculogen
1	*Alternaria arborescens*	**<0.5**	**<0.5**	**<0.5**	**<0.5**	**598 ± 11**	**883 ± 19**
2	*Alternaria astroemeriae*	**1.7 ± 0.1**	**1.8 ± 0.11**	**<0.5**	**<0.5**	**5.3 ± 0.1**	**<5**
3	*Aspergillus fumigatus*	**<0.5**	**<0.5**	**<0.5**	**<0.5**	**<1**	**1243 ± 25**
4	*Aspergillus niger*	**<0.5**	**<0.5**	**<0.5**	**<0.5**	**<1**	**1285 ± 26**
5	*Aspergillus pseudodeflectus*	**<0.5**	**<0.5**	**<0.5**	**<0.5**	**<1**	**1135 ± 25**
6	*Aureobasidium pullulans*	**<0.5**	**<0.5**	**<0.5**	**<0.5**	**25 ± 0.5**	**711 ± 31**
7	*Cladosporium cladosporioides*	**<0.5**	**<0.5**	**<0.5**	**<0.5**	**<1**	**133 ± 3**
8	*Cladosporium herbarum*	**<0.5**	**<0.5**	**<0.5**	**<0.5**	**1203 ± 24**	**276 ± 6**
9	*Dendrophoma juglandina*	**<0.5**	**<0.5**	**<0.5**	**<0.5**	**<1**	**124 ± 3**
10	*Diatrype stigma*	**3.6 ± 0.3**	**0.9 ± 0.06**	**<0.5**	**<0.5**	**<1**	**360 ± 7**
11	*Didymella negriana*	**<0.5**	**<0.5**	**<0.5**	**<0.5**	**7.4 ± 0.1**	**155 ± 4**
12	*Didymella sancta*	**<0.5**	**<0.5**	**24 ± 1.1**	**6.5 ± 0.3**	**12 ± 0.2**	**849 ± 18**
13	*Epicoccum nigrum*	**3.8 ± 0.3**	**<0.5**	**<0.5**	**<0.5**	**<1**	**<5**
14	*Lophiostoma corticola*	**<0.1**	**<0.5**	**<0.5**	**<0.5**	**<1**	**89 ± 2**
15	*Neosetophoma shoemakeri*	**<0.1**	**<0.5**	**<0.5**	**<0.5**	**<1**	**31.9 ± 0.7**
16	*Penicillium crustosum*	**<0.1**	**<0.5**	**0.9 ± 0.05**	**1.4 ± 0.06**	**338 ± 7**	**1187 ± 25**
17	*Phaeosphaeriaceae* sp.	**<0.1**	**<0.5**	**<0.5**	**<0.5**	**<1**	**136 ± 3**
18	*Pleurophoma ossicola*	**<0.1**	**<0.5**	**<0.5**	**<0.5**	**3585 ± 72**	**87 ± 2**
19	*Pseudogymnoascus pannorum*	**<0.1**	**<0.5**	**<0.5**	**<0.5**	**<1**	**206 ± 5**
20	*Sporocadus rosigena*	**<0.1**	**<0.5**	**<0.5**	**<0.5**	**<1**	**689 ± 15**

sd = standard deviation representing the repeatability of the analytical method used.

## Data Availability

The data presented in this study are available in supplementary material.

## Supplementary Materials

The following supporting information can be downloaded at https://www.mdpi.com/article/10.3390/toxins14020066/s1, Table S1: Overview of certified mycotoxin standards; Table S2: Overview of retention times and exact masses (m/z) of precursor ions and fragments of mycotoxins, together with normalized collision energies (NCE); precursor ions for fragmentation are highlighted.
