# Supplementary material for: Fungal Endophytes of Vitis vinifera—Plant Growth Promoters or Potentially Toxinogenic Agents?
Source: Toxins (Basel). 2022 Jan 19;14(2):66. doi: 10.3390/toxins14020066 (PMC8877596; doi:10.3390/toxins14020066)
Supplement: Supplementary file 1 [file toxins-14-00066-s001.zip › toxins-1531750-supplementary.pdf]

toxins

MDPI

Article

Fungal Endophytes of *Vitis vinifera* – Plant Growth Promoters or Potentially Toxinogenic Agents?

Milena Stranska, Zbynek Dzuman, Nela Prusova, Adam Behner, Irena Kolouchova, Petra Lovecka, Tomas Rezanka, Miroslav Kolarik and Jana Hajslova

Table S1. Overview of certified mycotoxin standards.

No.	Mycotoxin	CAS	Producer
1	15-Acetyldeoxynivalenol	88337-96-6	Romer Labs
2	3-Acetyldeoxynivalenol	50722-38-8	Romer Labs
3	Aflatoxin B1	1162-65-8	Merck
4	Aflatoxin B2	7220-81-7	Merck
5	Aflatoxin G1	1165-39-5	Merck
6	Aflatoxin G2	7241-98-7	Merck
7	Agroclavine	548-42-5	Romer Labs
8	Alternariol	641-38-3	Merck
9	Alternariol methyl ether	23452-05-3	Merck
10	Beauvericin	26048-05-5	Merck
11	Citrinin	518_75-2	Merck
12	Cyclopiazonic acid	18172-33-3	Merck
13	Deoxynivalenol	51481-10-8	Merck
14	Diacetoxyscirpenol	2270-40-8	Merck
15	Deoxynivalenol-3-glucoside	131180-21-7	Romer Labs
16	Enniatin A	2503-13-1	Merck
17	Enniatin A1	4530-21-6	Merck
18	Enniatin B	917-13-5	Merck
19	Enniatin B1	19914-20-6	Merck
20	Ergocornine	57432-60-7	Romer Labs
21	Ergocorninine	564-37-4	Romer Labs
22	Ergocristine	511-08-0	Romer Labs
23	Ergocristinine	511-07-9	Romer Labs
24	Ergocryptine	2706-66-3	Romer Labs
25	Ergocryptinine	511-10-4	Romer Labs
26	Ergometrine	60-79-7	Romer Labs
27	Ergosine	561-94-4	Romer Labs
28	Ergosinine	596-88-3	Romer Labs
29	Ergotamine	113-15-5	Romer Labs
30	Ergotaminine	639-81-6	Romer Labs
31	Fumonisin B1	116355-83-0	Merck
32	Fumonisin B2	116355-84-1	Merck
33	Fumonisin B3	136379-59-4	Romer Labs
34	Fusarenon X	23255-69-8	Merck
35	Gliotoxin	67-99-2	Merck
36	HT-2 toxin	26934-87-2	Romer Labs
37	Meleagrin	71751-77-4	Romer Labs
38	Mycophenolic acid	24280-93-1	Merck
39	Neosolaniol	36519-25-2	Merck

No.	Mycotoxin	CAS	Producer
40	Nivalenol	23282-20-4	Romer Labs
41	Ochratoxin A	303-47-9	Merck
42	Patulin	149-29-1	Merck
43	Paxilline	57186-25-1	Merck
44	Penicillic acid	90-65-3	Merck
45	Penitrem A	12627-35-9	Merck
46	Phomopsin A	64925-80-0	Enzo Life Sciences
47	Roquefortine C	58735-64-1	Merck
48	Stachybotrylactam	163391-76-2	Romer Labs
49	Sterigmatocystin	10048-13-2	Merck
50	T-2 toxin	21259-20-1	Romer Labs
51	Tenuazonic acid	610-88-8	Romer Labs
52	Tentoxin	28540-82-1	Romer Labs
53	Verrucarol	2198-92-7	Merck
54	Verruculogen	12771-72-1	Romer Labs
55	Zearalenone	17924-92-4	Romer Labs
56	α-zearalenol	364-55-72-8	Merck
57	β-zearalenol	71030-11-0	Merck

Table S2. Overview of retention times and exact masses (m/z) of precursor ions and fragments of mycotoxins, together with normalized collision energies (NCE); precursor ions for fragmentation are highlighted.

No.	Mycotoxin	Summary formula	RT (min)	ESI(−)		ESI(+)		NCE (%)	Fragment 1		Fragment 2	
				[M-H]$^-$	[M+CH_3COO]$^-$	[M+H]$^+$	[M+NH_4]$^+$		Summary formula	m/z	Summary formula	m/z
1	15-Acetyldeoxynivalenol	C17H22O7	2.69	337.1293	397.1504	339.1438	356.1704	10	C17H23O7	339.1438	C17H21O6	321.1333
2	3-Acetyldeoxynivalenol*	C17H22O7	2.57	337.1293	397.1504	339.1438	356.1704	10	C2H3O2	59.0138	C17H21O7	337.1292
3	Aflatoxin B1	C17H12O6	3.45	311.0561	371.0772	313.0707	330.0972	60	C14H9O4	241a.0495	C15H9O5	269.0444
4	Aflatoxin B2	C17H14O6	3.26	313.0718	373.0929	315.0863	332.1129	70	C14H11O5	259.0601	C14H11O4	243.0652
5	Aflatoxin G1	C17H12O7	3.01	327.0510	387.0722	329.0656	346.0921	60	C13H11O3	215.0703	C14H11O4	243.0652
6	Aflatoxin G2	C17H14O7	2.85	329.0667	389.0878	331.0812	348.1078	60	C14H13O4	245.0808	C13H13O3	217.0859
7	Agroclavine	C16H18N2	2.36	237.1397	297.1609	239.1543	256.1808	50	C15H14N	208.1121	C12H11N2	183.0917
8	Alternariol*	C14H10O5	3.41	257.0455	317.0667	259.0601	276.0866	70	C12H7O4	215.0350	C9H7O2	147.0452
9	Alternariol methyl ether*	C15H12O5	4.03	271.0612	331.0823	273.0758	290.1023	60	C14H8O5	256.0377	C13H8O4	228.0428
10	Beauvericin	C45H57N3O9	7.98	782.4022	842.4233	784.4168	801.4433	30	C15H18O2N	244.1332	C9H12N	134.0964
11	Citrinin*	C13H14O5	2.87	249.0768	309.0980	251.0914	268.1179	50	C13H13O4	233.0808	C13H15O5	251.0914

No.	Mycotoxin	Summary formula	RT (min)	ESI(–)		ESI(+)		NCE (%)	Fragment 1		Fragment 2	
				$[M-H]^-$	$[M+CH_3COO]^-$	$[M+H]^+$	$[M+NH_4]^+$		Summary formula	m/z	Summary formula	m/z
12	Cyclopiazonic acid*	C20H20N2O3	3.06	335.1401	395.1612	337.1547	354.1812	50	C6H6O3N	140.0353	C11H8N	154.0662
13	Deoxynivalenol*	C15H20O6	2.07	295.1187	355.1398	297.1333	314.1598	10	C2H3O2	59.0139	C15H19O6	295.1187
14	Deoxynivalenol-3-glucoside*	C21H30O11	1.98	457.1715	517.1927	459.1861	476.2126	20	C20H27O10	427.1610	C21H29O11	457.1715
15	Diacetoxyscirpenol	C19H26O7	3.53	365.1606	425.1817	367.1751	384.2017	20	C17H23O5	307.1540	C15H17O2	229.1223
16	Enniatin A	C36H63N3O9	8.36	680.4492	740.4703	682.4637	699.4903	20	C12H20O2N	210.1489	C36H64O9N3	682.4637
17	Enniatin A1	C35H61N3O9	8.20	666.4335	726.4546	668.4481	685.4746	20	C35H62O9N3	668.4481	C12H20O2N	210.1489
18	Enniatin B	C33H57N3O9	7.83	638.4022	698.4233	640.4168	657.4433	30	C11H18O2N	196.1332	C11H20O3N	214.1438
19	Enniatin B1	C34H59N3O9	8.03	652.4179	712.4390	654.4324	671.4590	30	C34H60O9N3	654.4324	C11H18O2N	196.1332
20	Ergocornine	C31H39N5O5	3.27	560.2878	620.3090	562.3024	579.3289	30	C16H18ON3	268.1444	C15H15N2	223.1230
21	Ergocorninine	C31H39N5O5	3.81	560.2878	620.3090	562.3024	579.3289	30	C19H17O2N2	305.1285	C15H15N2	223.1230
22	Ergocristine	C35H39N5O5	3.72	608.2878	668.3090	610.3024	627.3289	30	C15H15N2	223.1230	C16H18N3O	268.1444
23	Ergocristinine	C35H39N5O5	4.25	608.2878	668.3090	610.3024	627.3289	30	C15H15N2	223.1230	C19H17O2N2	305.1285
24	Ergocryptine	C32H41N5O5	3.67	574.3035	634.3246	576.3180	593.3446	30	C16H18N3O	268.1444	C15H15N2	223.1230
25	Ergocryptinine	C32H41N5O5	4.14	574.3035	634.3246	576.3180	593.3446	30	C15H15N2	223.1230	C17H19N2O2	283.1441
26	Ergometrine	C19H23N3O2	1.87	324.1718	384.1929	326.1863	343.2129	50	C14H10NO	208.0757	C15H15N2	223.1230
27	Ergosine	C30H37N5O5	3.01	546.2722	606.2933	548.2867	565.3133	30	C15H15N2	223.1230	C16H18N3O	268.1444
28	Ergosinine	C30H37N5O5	3.10	546.2722	606.2933	548.2867	565.3133	30	C15H15N2	223.1230	C16H18N3O	268.1444
29	Ergotamine	C33H35N5O5	3.15	580.2565	640.2777	582.2711	599.2976	30	C15H15N2	223.1230	C17H17N2O3	297.1234
30	Ergotaminine	C33H35N5O5	3.20	580.2565	640.2777	582.2711	599.2976	30	C15H15N2	223.1230	C14H10NO	208.0757
31	Fumonisin B1	C34H59NO15	4.37	720.3812	780.4023	722.3957	739.4223	30	C22H40ON	334.3104	C22H44O3N	370.3316
32	Fumonisin B2	C34H59NO14	5.46	704.3863	764.4074	706.4008	723.4274	30	C22H42ON	336.3261	C22H40N	318.3155
33	Fumonisin B3	C34H59NO14	4.99	704.3863	764.4074	706.4008	723.4274	30	C22H42ON	336.3261	C22H44O2N	354.3367
34	Fusarenon X*	C17H22O8	2.23	353.1242	413.1453	355.1387	372.1653	10	C2H3O2	59.0139	C17H21O8	353.1242
35	Gliotoxin*	C13H14N2O4S2	3.09	325.0322	385.0534	327.0468	344.0733	10	C2H6O2NS	108.0114	C5H5O4NS	174.9934

No.	Mycotoxin	Summary formula	RT (min)	ESI(−)		ESI(+)		NCE (%)	Fragment 1		Fragment 2	
				[M-H]$^-$	[M+CH$_3$COO]$^-$	[M+H]$^+$	[M+NH$_4$]$^+$		Summary formula	m/z	Summary formula	m/z
36	HT-2 toxin	C22H32O8	4.27	423.2024	483.2236	425.2170	442.2435	10	C15H19O4	263.1278	C14H15O2	215.1067
37	Meleagrin	C23H23N5O4	3.44	432.1677	492.1889	434.1823	451.2088	30	C17H12O3N5	334.0935	C22H21O3N5	403.1639
38	Mycophenolic acid	C17H20O6	4.68	319.1187	379.1398	321.1333	338.1598	50	C11H11O4	207.0652	C10H9O3	177.0546
39	Neosolaniol	C19H26O8	2.30	381.1555	441.1766	383.1700	400.1966	10	C17H21O5	305.1384	C15H17O3	245.1172
40	Nivalenol*	C15H20O7	1.82	311.1136	371.1348	313.1282	330.1547	10	C2H3O2	59.0139	C14H17O6	281.1031
41	Ochratoxin A	C20H18ClNO6	5.34	402.0750	462.0961	404.0895	421.1161	20	C19H17O4NCl	358.0841	C11H8O4Cl	239.0106
42	Patulin*	C7H6O4	1.82	153.0193	213.0405	155.0339	172.0604	30	C6H5O2	109.0295	C5H5O	81.0346
43	Paxilline	C27H33NO4	6.93	434.2337	494.2548	436.2482	453.2748	20	C27H32O3N	418.2377	C9H8N	130.0651
44	Penicillic acid	C8H10O4	2.08	169.0506	229.0718	171.0652	188.0917	40	C7H9O2	125.0597	C6H9O	97.0648
45	Penitrem A*	C37H44NO6Cl	4.42	632.2784	692.2996	634.2930	651.3195	40	C32H33O5NCl	546.2053	C26H23O2NCl	416.1423
46	Phomopsin A	C36H45ClN6O12	2.60	787.2711	847.2923	789.2857	806.3122	10	C12H15O5N3	281.1006	C9H18O8N2	282.1058
47	Roquefortine C	C22H23N5O2	4.22	388.1779	448.1990	390.1925	407.2190	40	C8H9O2N4	193.0720	C17H16O2N5	322.1299
48	Stachybotrylactam	C23H31NO4	6.31	384.2180	444.2392	386.2326	403.2591	60	C9H8O3N	178.0499	C8H8O2N	150.0550
49	Sterigmatocystin	C18H12O6	5.68	323.0561	383.0772	325.0707	342.0972	50	C17H10O6	310.0472	C16H9O5	281.0444
50	T-2 toxin	C24H34O9	4.89	465.2130	525.2341	467.2276	484.2541	10	C21H31O7	395.2064	C12H17O4	225.1121
51	Tentoxin	C22H30N4O4	4.58	413.2194	473.2406	415.2340	432.2605	30	C18H22O2N3	312.1707	C9H19ON2	171.1492
52	Tenuazonic acid	C10H15NO3	3.74	196.0979	256.1190	198.1125	215.1390	45	C6H5O3	125.0233	C9H13O2	153.0910
53	Verrucarol	C15H22O4	2.53	265.1445	325.1657	267.1591	284.1856	10	C15H21O3	249.1485	C15H19O2	231.1380
54	Verruculogen	C27H33N3O7	6.09	510.2246	570.2457	512.2391	529.2657	20	C19H18O4N3	352.1292	C19H20O5N3	370.1397
55	Zearalenone*	C18H22O5	3.85	317.1394	377.1606	319.1540	336.1805	50	C9H7O	131.0502	C10H7O3	175.0401
56	α-zearalenol*	C18H24O5	3.75	319.1551	379.1762	321.1697	338.1962	60	C9H4O3	160.0166	C9H6O	130.0424
57	β-zearalenol*	C18H24O5	3.53	319.1551	379.1762	321.1697	338.1962	60	C9H6O	130.0424	C9H4O3	160.0166

* Fragment ions are sorted according to decreasing signal, fragment 1 was thus used for quantification for analytes determined in paralell reaction monitoring (PRM) mode (ESI−).
